# Selection of Reliable Reference Genes for RT-qPCR Analysis of *Bursaphelenchus mucronatus* Gene Expression From Different Habitats and Developmental Stages

**DOI:** 10.3389/fgene.2018.00269

**Published:** 2018-07-23

**Authors:** Lifeng Zhou, Fengmao Chen, Jianren Ye, Hongyang Pan

**Affiliations:** Collaborative Innovation Center of Sustainable Forestry in Southern China, College of Forestry, Nanjing Forestry University, Nanjing, China

**Keywords:** *Bursaphelenchus mucronatus*, reference gene, normalization, RT-qPCR, validation

## Abstract

Quantitative reverse transcription polymerase chain reaction (RT-qPCR), a sensitive technique for gene expression analysis, depends on the stability of the reference genes used for data normalization under different experimental conditions. *Bursaphelenchus mucronatus*, a pine-parasitic nematode varying in virulence, is widely distributed in natural pine forests throughout the northern hemisphere, but has not been investigated with respect to the identification of reference genes suitable for the normalization of RT-qPCR data. In the present study, eight candidate reference genes were analyzed in *B. mucronatus* under different habitat conditions and at different developmental stages. The expression stability of these genes was assessed by geNorm, NormFinder, BestKeeper, delta Cq, and RefFinder algorithms. In general, our results identified encoding beta-tubulin as the most stable gene. Moreover, pairwise analysis showed that three reference genes were sufficient to normalize the gene expression data under each set of conditions, with genes encoding beta-tubulin, 18S ribosomal RNA and ubiquitin-conjugating enzyme being the most suitable reference genes for different habitat conditions, whereas genes encoding beta-tubulin, histone, and 18S ribosomal RNA exhibited the most stable expression at different developmental stages. Validation of the selected reference genes was performed by profiling the expression of the fatty acid- and retinol-binding protein gene in different habitats, and by profiling the expression of the arginine kinase gene at different developmental stages. This first systematic analysis for the selection of suitable reference genes for RT-qPCR in *B. mucronatus* will facilitate future functional analyses and deep mining of genetic resources in this nematode.

## Introduction

Quantitative reverse transcription polymerase chain reaction (RT-qPCR) is one of the most effective technologies used for quantifying gene expression in terms of transcript abundance and is characterized by high sensitivity and specificity, high reproducibility, and high-throughput capacity for a limited number of target genes (Heid et al., 1996; Livak and Schmittgen, 2001). RT-qPCR is a valuable method to analyze transcript abundance levels in different organisms (Giulietti et al., 2001) at different developmental stages (Huang et al., 2014; Wang et al., 2015) and under different physiological conditions. However, the accuracy of qPCR is influenced by a number of variables, such as RNA integrity, quantity, enzymatic efficiency in cDNA synthesis, and PCR amplification. Therefore, to avoid deviations in expression levels as a result of such technical factors, a normalization step is an essential precondition. The most widely used method for normalization is to include one or a series of reference genes, whose expression is presumed to be stable under different experimental conditions.

To obtain the available reference genes for qPCR, several statistical algorithms have been established to identify reference genes with stable expression levels, such as the delta Cq method, BestKeeper, geNorm, NormFinder, and RefFinder. The delta Cq method calculates the relative expression levels between a candidate reference gene and other candidate reference genes within each sample, and compares the standard deviation (SD) of delta Cq to estimate the most stable reference genes (Silver et al., 2006). The geNorm algorithm evaluates the most stable reference genes by sequential elimination of the highest *M*-value (MV) reference genes, and determines the optimal number of reference genes required for normalization by calculating pairwise variation between each reference gene and the other reference genes (Vandesompele et al., 2002). NormFinder ranks candidate reference genes by calculating their stability value (SV) among samples in the given groups, and selects genes with a lower SV, considered to exhibit higher expression stability (Andersen et al., 2004). The BestKeeper algorithm calculates the stability of the candidate genes based on the SD of their *C*q values, plus the coefficient of variance (CV), correlation coefficient (*r*), and the *p*-value (p) which are also very important parameters (Pfaffl et al., 2004). The RefFinder software comprehensively ranks the candidate reference genes based on the geometric mean (GM) values of the results from the four different statistical algorithms described above (Xie et al., 2012). With the help of these five statistical algorithms, much research on the validation of reference genes under different experimental conditions has been reported (Liu et al., 2017; Sadangi et al., 2017; Sprang et al., 2017; Mughal et al., 2018).

*Bursaphelenchus mucronatus* Mamiya & Enda is a migratory endoparasitic nematode that infects coniferous trees, is a sister species to *Bursaphelenchus xylophilus* (Steiner & Buhrer) Nickle, the causative agent of pine wilt disease, and is widely distributed in natural pine forests throughout the northern hemisphere (Mamiya and Enda, 1979; Kulinich et al., 1994; Pereira et al., 2013). The morphology and life history of *B. mucronatus* is very similar to that of *B. xylophilus*, including its host range of conifer species and the phoretic relationship with cerambycid beetles (Mamiya and Enda, 1979). The life cycle of *B. mucronatus* is composed of five stages, namely, eggs, second-stage juveniles (J2), third-stage juveniles (J3), fourth-stage juveniles (J4), and adults (Mamiya and Enda, 1979; Wang et al., 2005). *B. mucronatus* was initially considered to be non-pathogenic, or pathogenic (with a very low virulence level) only under conditions of high temperature and drought, although it had been reported to have killed some pine seedlings (Mamiya and Enda, 1979; Futai, 1980b; Tomminen, 1993; Braasch, 1996). In recent years, however, research has shown that the virulence of *B. mucronatus* varies to some extent. For example, *B. mucronatus* isolates from Asia and Europe were highly virulent to pine seedlings and trees under greenhouse or outdoor conditions (Akbulut et al., 2007; Dayi and Akbulut, 2012; Akbulut et al., 2014; Zhou L.F. et al., 2016).

With the development of biotechnology, increasing biological data of *B. mucronatus* including transcriptome (Yan et al., 2012; Zhou L. et al., 2016) and proteome (Cardoso et al., 2016; Zhou L. et al., 2016) were available. Furthermore, the expression profiles and functions of some genes in this nematode had been investigated, such as heat shock protein genes (Huang et al., 2009; Dai et al., 2011), cathepsin gene (Pan et al., 2015), fatty acid- and retinol-binding protein gene (Zhou L. et al., 2016), expansin gene (Zhou L. et al., 2016), cellulase gene (Wang et al., 2017), and so on. It shows urgent need to select reference genes for *B. mucronatus* under different experimental conditions. Hence, in the present study, eight genes commonly used for transcript normalization were selected as candidate reference genes and their expression stabilities in *B. mucronatus* were evaluated by the five statistical methods. Seven of the genes are protein-coding genes, namely actin (*ACT*), elongation factor 1-alpha (*EF*), histone (*HIS*), peroxisomal membrane protein (*PMP*), beta-tubulin (*TUB*), ubiquitin-conjugating enzyme (*UBCE*), and ubiquitin (*UB*Q). The remaining candidate gene is the non-protein-encoding gene 18S ribosomal RNA (*18S rRNA*), which is widely used in gene expression studies. Different algorithms and statistical analyses were employed to evaluate the expression stability of the candidate reference genes in *B. mucronatus* in different habitats (fungus and trees) and at key developmental stages (eggs, larvae, and adults). Additionally, the candidate reference genes were used to profile the expression of target genes in *B. mucronatus* under different habitat conditions and different developmental stages, to validate the results obtained. This work will benefit future studies on gene expression in *B. mucronatus*, speed up functional analyses of special genetic resources, and improve our understanding of the molecular characteristics of this nematode and other *Bursaphelenchus* species.

## Materials and Methods

### Nematodes Material

The *B. mucronatus* isolate used in the experiments in the current study was originally isolated from wilted pine trees in Jiangsu province of China during a field survey in 2009, and since then was maintained on fungal cultures of *Botrytis cinerea* Pers. on potato-dextrose agar (PDA) plates at the Institute of Forest Protection Lab in Nanjing Forestry University (Nanjing, China). *B. mucronatus* was reared on cultures of *B. cinerea* growing on PDA plates at 25°C for 7 days and isolated using a modified Baermann funnel technique for 2 h at 25°C (Viglierchio and Schmitt, 1983) and cleaned using sucrose flotation and phosphate-buffered saline Tween 20 (PBST) (Kikuchi et al., 2011).

### Collection of Eggs

The eggs were collected from the cleaned nematodes as reported in previous research (Futai, 1980a; Wang et al., 2005). A proportion of the collected eggs were immediately frozen in liquid nitrogen and stored at –80°C, while the others were used in subsequent experiments.

### Collection of Second-Stages Juveniles

The collected eggs were suspended in distilled water, added to Petri dishes, and incubated at 25°C in the dark. The J2 were collected by decanting the supernatant into new Petri dishes, with the unhatched eggs remaining in the bottom of the dishes due to their sticky nature (Futai, 1980a). By carrying out the collection process every 2 h for 15–20 times, most of the J2 were collected. Subsequently, non-J2 were removed under a compound microscope at 100× magnification (Zeiss MicroImaging GmbH, Oberkochen, Germany). A portion of the collected J2 was immediately frozen in liquid nitrogen and stored at –80°C, while the remaining J2 were used in subsequent experiments.

### Collection of Mix Third- and Fourth-Stage Juveniles

The collected J2 were reared on cultures of *B. cinerea* growing on PDA plates at 25°C for ∼40 h and isolated using a modified Baermann funnel technique for 2 h at 25°C and cleaned as previously described. Then non-J3&4 were removed under a compound microscope at 100× magnification (Zeiss MicroImaging GmbH). The collected J3&4 were immediately frozen in liquid nitrogen and stored at –80°C.

### Collection of Adults Nematodes

The collected J2 were reared on cultures of *B. cinerea* growing on PDA plates at 25°C for ∼5 days and isolated using a modified Baermann funnel technique for 2 h at 25°C and cleaned as previously described. Then non-adult nematodes were removed under a compound microscope at 100× magnification (Zeiss MicroImaging GmbH). The collected adults were immediately frozen in liquid nitrogen and stored at –80°C.

### Collection of Nematodes During the Pathogenic Process

The *B. mucronatus* nematodes were inoculated onto *Pinus thunbergii* Parl. trees as reported in previous research (Zhou L.F. et al., 2016). The nematodes were extracted from the wilted trees using a modified Baermann funnel technique and cleaned as previously described, and then promptly frozen in liquid nitrogen and stored at –80°C. The re-isolated nematodes were identified based on their morphological and molecular characteristics (Burgermeister et al., 2005; Kanzaki et al., 2006).

### Total RNA Isolation and cDNA Synthesis

Total RNA from each sample was extracted using the RNAprep Kit (Tiangen, Beijing, China) and then purified further with the RNAclean Kit (Tiangen), following the manufacturer’s instructions. Total RNA was quantified with an Agilent 2100 Bioanalyzer RNA Nanochip (Agilent Technologies, Inc., Waldbronn, Germany), and RNA integrity was verified by agarose gel electrophoresis. Reverse transcription was carried out using TransScript^®^ One-Step gDNA Removal and cDNA Synthesis SuperMix Kit (Transgen), following the manufacturer’s instructions.

### Selection of Candidate Reference Genes and qPCR Primer Design

Eight candidate reference genes were selected which had previously been reported to be suited to transcript normalization in other organisms under different experimental conditions. The sequences of candidate reference genes were obtained from the transcriptome *de novo* assembly sequences of *B. mucronatus* (Zhou L. et al., 2016). Primers were designed using Primer Premier 5 software (Premier Biosoft International, Palo Alto, CA, United States), according to the SYBR Green Master Mix (Vazyme, Nanjing, China) manufacturer’s instructions.

### RT-qPCR

RT-qPCR was carried out using the StepOne Plus Real-time PCR System (Applied Biosystems, Foster City, CA, United States), in which amplification, detection, and analysis steps were combined. Reactions were performed using the SYBR Green Master Mix (Vazyme, Nanjing, China) in a 20 μL reaction volume, containing 10 μL SYBR Green Master Mix, 0.4 μL 10 pmol/L of each primer, and 2 μL diluted cDNA. The following program parameters were used for all amplifications: 95°C for 5 min, followed by 40 cycles at 95°C for 15 s each, 60°C for 32 s, and finally one cycle at 95°C for 15 s, 60°C for 1 min, 95°C for 15 s, and 60°C for 15 s to generate dissociation curves. The amplification efficiency (E) was calculated from standard curves, according to the SYBR Green Master Mix manufacturer’s instructions. All assays were performed using three biological replicates, each consisting of technical triplicates, and a non-template control.

### Assessing the Expression Stability of Reference Genes

The expression stabilities of the candidate reference gene candidates were estimated and ranked using four different statistical algorithms, geNorm, NormFinder, BestKeeper, and the delta Cq method, and a web-based analysis tool, RefFinder. The geNorm evaluates the most stable reference genes and determines the optimal number of reference genes required for normalization (Vandesompele et al., 2002). The NormFinder ranks candidate reference genes by calculating their SV (Andersen et al., 2004). The BestKeeper calculates the stability of the candidate genes based on the SD of their *C*q values (Pfaffl et al., 2004). The comparative delta *C*q method calculates the delta *C*q of the candidate reference genes (Silver et al., 2006). Finally, we comprehensively ranked the candidate reference genes based on the GM values of the above results from the four different statistical algorithms, using the web-based analysis tool RefFinder^1^ (Xie et al., 2012).

### Validation of Reference Genes

To confirm the stability of expression of the selected reference genes, verification experiments were carried out in samples from different habitats and different developmental stages. For the habitat conditions, we quantified the relative expression of the fatty acid- and retinol- binding protein gene (*FAR*), which functions to facilitate parasitic nematode infections (Fairfax et al., 2009). For the developmental stage studies, we quantified the relative expression of arginine kinase genes (*AK1* and *AK2*), which have been shown to be differentially expressed at different developmental stages (Kulathunga et al., 2012; Qi et al., 2015). Normalization of the two target genes was conducted using the most stable gene combinations (*TUB*/*18S rRNA*/*UBCE* or *TUB*/*HIS*/*18S rRNA*) and the least stable combinations (*EF/PMP/ACT* or *ACT/EF/PMP*) as determined by geNorm and RefFinder. Relative quantification of these two target genes was calculated using the 2^-ΔΔCt^ method (Livak and Schmittgen, 2001). The primers for validation of selected reference genes were as follows: *FAR*-F, 5′-CACGGGCTTGTTTATTGACC-3′ and *FAR*-R, 5′-CACTCAAACTGCAGCCACAT-3′; *AK1*-F, 5′-TTGGTTGGCTGACCTTCTG-3′ and *AK1*-R, 5′-CGAGTGCTCACCGTGGAT-3′; and *AK2*-F, 5′-CGGACGCCGAGTCTTACA-3′ and *AK2*-R, 5′-GCCAAGATCAACGGGAGG-3′. Statistical analysis was performed by SPSS 18.0 (SPSS Inc., Chicago, IL, United States) based on the independent sample *t*-test.

## Results

### Identification of *B. mucronatus* Candidate Reference Genes

Based on the transcriptome *de novo* assembly sequences of *B. mucronatus* (Supplementary Table S1), two to three primer pairs were designed for each candidate reference gene to amplify one single PCR product, and confirmed by the dissociation assay following RT-qPCR. The primer pair with a single peak in the melting curve analyses indicated high specificity (Supplementary Figure S1). Additionally, the amplification efficiency (E) of the single-peak primers was determined by serial decimal dilution of the cDNA solution and subsequently plotting the *C*q values versus the logarithm-transformed initial template concentration (Supplementary Figure S2). For each candidate reference gene, the primer pair with a single melt curve peak and the greatest efficiency was retained (**Table 1**). The amplification efficiencies of the candidate reference genes ranged from 93.5 to 99.3% and all the correlation coefficients (*r*^2^) were greater than 0.99.

**Table 1:** Primer sequences and amplification efficiency of candidate reference genes.

Gene symbol	Primer sequence (5′ to 3′)	Amplicon length (bp)	*E* (%)	*r*^2^
*ACT*	F: CTCCCAGCCGAACTAACAAACC	178	95.3	0.999
	R: CTTCTGTCCCATACCGACCATG			
*EF*	F: TCAGGCTGATTGTGCTGTCTTG	199	96.7	0.999
	R: TTCGTTGACCACTTCGGTGTAA			
*HIS*	F: GCCATTCGTCGTCTCGCTCGTC	106	95.9	0.999
	R: CACGGATCACATTCTCCAAGAAC			
*PMP*	F: TGGAGGACGGAAGATACGAG	176	95.3	0.999
	R: TTGGGAGTGACCAGTGGAAC			
*TUB*	F: TGACAACGAAGCCCTTTACGAC	200	99.3	0.999
	R: CAGGCATGAAGAAGTGGAGACG			
*UBCE*	F: ATCGCCTACGGTCTACGGGTAC	124	95.9	0.999
	R: TAACGGCAGATAAATTGCTGGA			
*UBQ*	F: CTTCGTCTCCGTGGAGGTAT	109	96.7	0.999
	R: GGATCTTGGCTTTGACATTCT			
*18S rRNA*	F: TTCTGACCGTAAACGATGCCAACT	169	93.5	0.999
	R: ATTAAGCCGCAAGCTCCACTCC			


### Expression Levels of Candidate Reference Genes

**FIGURE 1:**
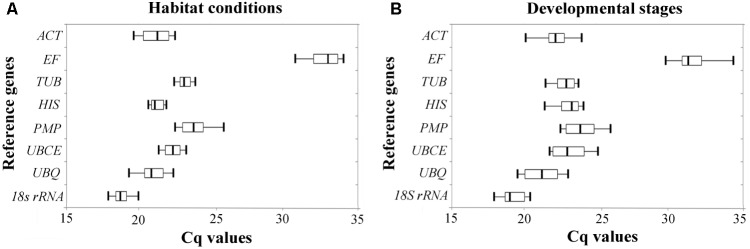
Expression level of candidate reference genes were determined by RT-qPCR under different **(A)** habitat conditions and **(B)** developmental stages. Three hundred nanograms of cDNA were used as template in each reaction. Each box plot of *C*q values is shown as the first and third quartile, and the median value is represented by the vertical line in the box. The whiskers show the maximal and minimal values.

The relative expression levels of the eight candidate reference genes were determined by their *C*q values in two experimental sets; two habitat conditions (**Figure 1A**), and four developmental stages (**Figure 1B**). The range of the *C*q values showed variability among the eight candidate reference genes, the lowest range of *C*q values under the two habitat conditions being from *HIS* and *TUB*, while the lowest range of *C*q values between the different developmental stages were from *TUB* and *18S rRNA*, indicating that these candidate genes are more stable than others. However, a simple comparison of the range of *C*q values is insufficient to evaluate the expression stability of the reference genes. Hence, we performed the following five statistical algorithms for verification.

### Determination of the Expression Stability of the Reference Genes Under Different Habitat Conditions

**FIGURE 2:**
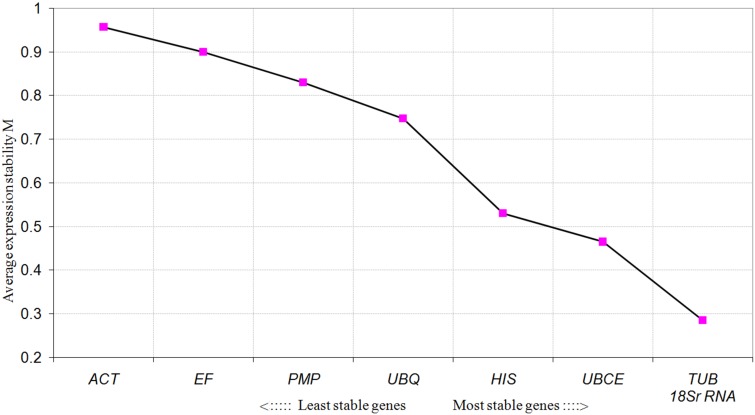
Average expression stability values (MV) of the candidate reference genes calculated by geNorm under different habitat conditions.

To further evaluate the stability of expression of the eight candidate reference genes under different habitat conditions, five widely used statistical algorithms (geNorm, NormFinder, BestKeeper, delta Cq method, and RefFinder) were employed (**Table 2**). The geNorm algorithm showed that all eight candidate genes were suitable as reference genes (MV < 1.5), with *TBU* and *18S rRNA* being the most stable genes (**Figure 2**). Similar results were obtained using NormFinder, which demonstrated that *18S rRNA* and *TBU* had the most stable expression. Additionally, these genes also showed the lowest intra-group variation (Supplementary Table S2), which was the key factor determining ranking in the NormFinder analysis. The results of the BestKeeper analysis showed that *HIS*, *TUB*, *18S rRNA* were the most stable genes, all with *SD* < 1 and *r* < 0.05, and thus qualifying as reference genes, whereas *ACT* was not suitable as a reference gene (*SD* > 1) (**Table 3**; Migocka and Papierniak, 2011). We also used the delta Cq algorithm to rank the expression stability of the eight candidate reference genes, under which *TBU* and *18S rRNA* were identified as the most stable genes (**Table 2**). Finally, mean *C*q values were entered into the RefFinder web page and, based on the rankings obtained for each gene using the previous four algorithms, the comprehensive ranking was obtained; *TUB*, *18S rRNA*, and *UBCE* proved to be the most stable genes, all with GM < 9 (**Table 2**).

**Table 2:** Expression stability ranking under different habitat conditions of the eight candidate reference genes using five algorithms.

Gene	RefFinder		geNorm		NormFinder		BestKeeper		Delta Cq	
	Rank	GM	Rank	MV	Rank	SV	Rank	*SD*	Rank	*SD*
*TUB*	1	1.41	1	0.72	2	0.09	2	0.43	1	0.69
*18S rRNA*	2	1.57	2	0.73	1	0.07	3	0.53	2	0.71
*UBCE*	3	3.22	3	0.97	3	0.44	4	0.59	3	0.89
*HIS*	4	3.31	5	1.01	5	0.50	1	0.32	5	0.98
*UBQ*	5	4.47	4	0.98	7	0.60	5	0.74	4	0.97
*EF*	6	6.19	6	1.03	4	0.45	7	0.97	6	1.01
*PMP*	7	6.48	7	1.09	8	0.82	6	0.92	7	1.03
*ACT*	8	8.00	8	1.13	6	0.57	8	1.04	8	1.06


*Rankings were determined using the parameters GM, geometric mean; SV, stability value; MV, *M*-Value; and SD, standard deviation.*

**Table 3:** Stability of the eight candidate genes under different habitat conditions of *B. mucronatus* based on *C*q values evaluated by BestKeeper software.

	*ACT*	*EF*	*TUB*	*HIS*	*PMP*	*UBCE*	*UBQ*	*18S rRNA*
*SD*	1.04	0.97	0.43	0.32	0.92	0.59	0.74	0.53
CV	4.90	2.94	1.85	1.48	3.85	2.62	3.54	2.81
*r*	0.772	0.831	0.953	0.181	0.746	0.521	0.727	0.968
*p*	0.001	0.001	0.001	0.001	0.001	0.001	0.001	0.001


*SD, standard deviation; CV, Coefficient of variation; r, coefficient of correlation; p, *p*-value.*

### Determination of the Expression Stability of the Reference Genes at Different Developmental Stages

**FIGURE 3:**
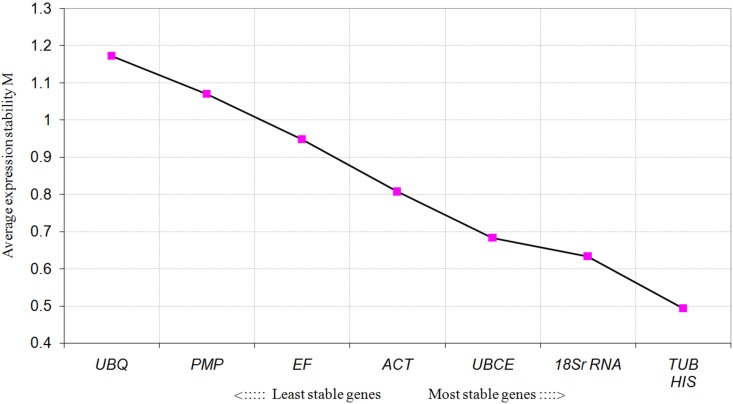
Average expression stability values (MV) of the candidate reference genes calculated by geNorm at different developmental stages.

For the different developmental stages, *TUB* and *HIS* were the most stable genes from the geNorm analyses, followed by *18S rRNA* (**Figure 3**). In the NormFinder analyses, the ranking of the eight candidate genes was similar, though not identical to that using geNorm (**Table 4**). The BestKeeper analysis identified the same three genes (*TBU*, *18S rRNA*, and *HIS*) as having the most stable expression, whereas expression of *ACT*, *UBCE*, and *PMP* were relatively unstable (*SD* > 1) (**Table 5**). The comprehensive ranking generated by RefFinder was consistent with the results of the delta *C*q algorithm, from most stable to least stable as follows: *TUB*, *HIS*, *18S rRNA*, *UBQ*, *UBCE*, *ACT*, *EF*, and *PMP* (**Table 4**).

**Table 4:** Stability of the eight candidate genes at different developmental stages of *B. mucronatus* based on *C*q values evaluated by BestKeeper software.

	*ACT*	*EF*	*TUB*	*HIS*	*PMP*	*UBCE*	*UBQ*	*18S rRNA*
*SD*	1.16	0.98	0.61	0.78	1.49	1.17	0.98	0.64
CV	5.11	3.12	2.70	3.42	6.13	5.08	4.60	3.30
*r*	0.746	0.690	0.987	0.931	0.411	0.990	0.986	0.853
*p*	0.001	0.001	0.001	0.001	0.001	0.001	0.001	0.001


*SD, standard deviation; CV, Coefficient of variation; r, coefficient of correlation; p, *p*-value.*

**Table 5:** Expression stability ranking at different developmental stages of the eight candidate reference genes using five algorithms.

Gene	RefFinder		geNorm		NormFinder		BestKeeper		Delta Cq	
	Rank	GM	Rank	MV	Rank	SV	Rank	*SD*	Rank	*SD*
*TUB*	1	1.00	1	0.88	2	0.17	1	0.61	1	0.80
*HIS*	2	1.86	2	0.92	1	0.09	3	0.78	2	0.83
*18S rRNA*	3	3.08	3	0.99	3	0.38	2	0.64	3	0.87
*UBQ*	4	3.94	8	1.48	8	0.91	4	0.98	4	0.88
*UBCE*	5	5.32	4	1.07	4	0.45	7	1.17	5	0.89
*ACT*	6	5.42	6	1.35	7	0.82	6	1.16	6	1.17
*EF*	7	7.00	7	1.39	6	0.81	5	0.99	7	1.28
*PMP*	8	7.44	5	1.32	5	0.73	8	1.49	8	1.34


*Parameters of rankings were determined using the parameters GM, geometric mean; SV, stability value; MV, *M*-Value; and SD, standard deviation.*

### Determination of the Optimal Number of Reference Genes for Reliable Normalization Under Different Habitat Conditions and Developmental Stages

**FIGURE 4:**
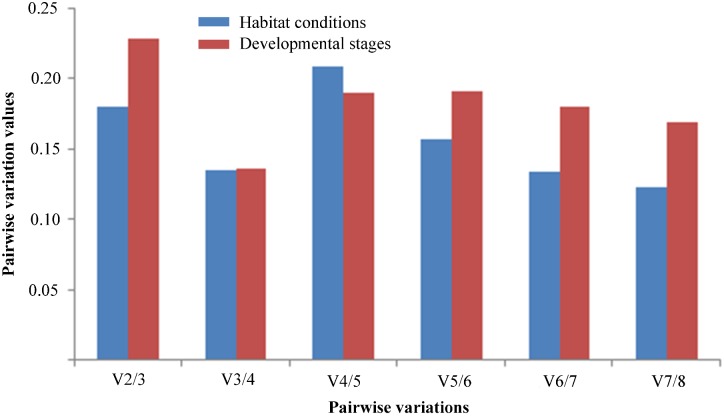
Pairwise variations (V) of the expression stability of candidate reference genes calculated by geNorm in two experimental sets. Pairwise variations (Vn/n + 1) were analyzed to determine the optimal number of the reference genes for normalizing RT-qPCR data.

To determine the optimal number of reference genes for normalization of RT-qPCR data, the pairwise variation value (Vn/n + 1) was calculated by geNorm. A threshold value below 0.15 suggests that the number of gene pairings will be sufficient for normalization (Vandesompele et al., 2002). Under different habitat conditions, V3/4 was lower than 0.15, suggesting that the optimal number of stable reference genes for normalization was three, namely *TUB*, *18S rRNA*, and *UBCE*. Similarly, at different developmental stages, V3/4 was lower than 0.15, indicating that three stable reference genes were suitable for normalization, namely *TUB*, *HIS*, and *18S rRNA* (**Figure 4**).

### Validation of the Proposed Reference Genes Under Different Experimental Conditions

**FIGURE 5:**
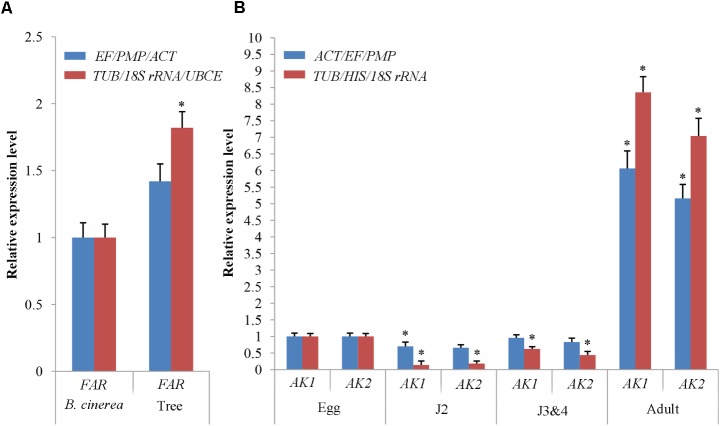
Evaluation of reference genes using **(A)** the fatty acid- and retinol- binding protein gene (*FAR*) under both habitat conditions and **(B)** the arginine kinase genes (*AK1* and *AK2*) in all developmental stages with the three most stable reference genes and the most unstable reference gene. The expression levels of *FAR* in *Botrytis cinerea* **(A)** and of *AK1*, *AK2* in the eggs **(B)** were set at a relative expression of 1 (control). Data points indicate the mean ± SE calculated from three biological replicates. The asterisks indicate statistically significant differences (*p* < 0.05) from the control.

To validate the selection of reference genes in *B. mucronatus* under different experimental conditions, we checked *FAR* expression under both habitat conditions (culture with tree or fungus), and *AK1* and *AK2* at all developmental stages following normalization with the proposed stable gene combinations (*TUB*, *18S rRNA*, and *UBCE* or *TUB*, *HIS*, and *18S rRNA*, respectively) and the least stable combinations (*EF/PMP/ACT* or *ACT/EF/PMP*, respectively) (**Figure 5**). Under different habitat conditions, *FAR* expression was found to be 1.82-fold (*p* < 0.05) higher from nematodes isolated from infected trees than in the fungal culture control when the proposed gene combination (*TUB*/*18S rRNA*/*UBCE*) was used. When the least stable combination (*EF/PMP/ACT*) was used, *FAR* expression was only 1.42-fold (*p* > 0.05) higher in nematodes extracted from trees than in the controls under the same conditions. At different developmental stages, *AK1* expression was sharply downregulated at J2 (0.14-fold) (*p* < 0.05) and J3&4 stages (0.62-fold) (*p* < 0.05), but was sharply upregulated at the adult stage (8.3-fold) (*p* < 0.05), when the proposed gene combination (*TUB*/*HIS/18S rRNA*) was used. Conversely, use of the least stable combination (*ACT/EF/PMP*) showed *AK1* expression to be similar in eggs and J3&4 (1- and 0.96-fold, respectively) (*p* > 0.05), but to be regulated at J2 and adult stages (0.70- and 6.1-fold, respectively) (*p* < 0.05). The expression trend of *AK2* was similar to that of *AK1*.

## Discussion

RT-qPCR is the technique most widely used for quantifying gene expression levels due to its high sensitivity and specificity, high reproducibility, and high-throughput capacity (Hellemans et al., 2007). A reliable RT-qPCR quantification assay needs suitable reference genes as internal controls, according to MIQE (Minimum information for publication of quantitative) guidelines (Bustin et al., 2009). Previous research had shown that no single reference gene could be effectively used in the quantification of gene expression levels in all species or under all experimental conditions, because its expression levels always vary considerably under different experimental conditions (Suzuki et al., 2000; Kozera and Rapacz, 2013). An effective reference gene should be stably expressed under the different experimental treatments being compared, while employing non-validated reference genes may result in incorrect conclusions. The present study is the first to evaluate and validate a series of candidate reference genes which are suitable for RT-qPCR gene expression analysis under different habitat conditions (fungus and trees) and different developmental stages (eggs, larvae, and adults) in *B. mucronatus*. *TUB*, *18S rRNA*, and *UBCE* could be applied as stable reference genes in future molecular studies that aim to understand the mechanisms of pathogenicity in *B. mucronatus*. The reliable combination *TUB*, *HIS*, and *18S rRNA* will help facilitate the molecular mechanisms conferring development and functional genomics studies of the nematode in future work.

Four different methods, geNorm, NormFinder, BestKeeper, and delta Cq, are widely used to identify stable reference genes. However, it is difficult to identify reference genes which are generally stable across these four algorithms, because each of them has its own strengths and appropriate application conditions (Vandesompele et al., 2002; Silver et al., 2006; Nakayama et al., 2018). Therefore, for many researchers, the selection of the optimum assessment methods is difficult. In addition, in cases where multiple methods are employed, a comprehensive ranking based on results from the different methods is necessary. In the current study, to overcome the variation in results from the different algorithms and to obtain a final ranking, a web-based analysis tool, RefFinder, was employed after evaluation by the four independent methods. The final consolidated and holistic ranking obtained from RefFinder was derived from the GM of the ranking values from the component four algorithms (Xie et al., 2012). A weakness of RefFinder is that the results of the four methods are not weighted on the basis of the inapplicability of their cut-offs and appropriate weights (Wu et al., 2014).

Based on the results from the comprehensive analysis using the five algorithms, *TUB*, *18S rRNA*, and *UBCE* were found to exhibit the most stable gene expression in *B. mucronatus* under the two habitat conditions, while *EF*, *PMP*, and *ACT* were the least stable. Not surprisingly, each algorithm gave a different ranking. According to previous research, the use of multiple reference genes is preferable to gain a more accurate and reliable result (Vandesompele et al., 2002; Martins et al., 2016; Liu et al., 2017). The optimal number of reference genes to achieve normalization can be calculated using geNorm, where the lowest number of gene combinations which causes the threshold value to fall below 0.15 indicates the number of reference genes which will be sufficient for normalization (Vandesompele et al., 2002). The geNorm algorithm calculated that the optimal number of reference genes for normalization under different habitat conditions was three, namely *TUB*, *18S rRNA*, and *UBCE*. For the different developmental stages in *B. mucronatus*, all five algorithms obtained the same three top-ranked genes with few differences in their respective orders. *TUB* was the most stable reference gene, followed by *HIS*, and *18S rRNA*, according to geNorm, delta Cq, and RefFinder. However, *HIS* was the most stable reference gene, followed by *TUB*, and *18S rRNA*, according to NormFinder, while *TUB* was the most stable gene, followed by *18S rRNA*, and *HIS*, according to BestKeeper. As with the two habitat conditions, three reference genes were sufficient for normalization at different developmental stages, according to geNorm.

*TUB* and *18S rRNA* were the most stable reference genes under different habitat conditions and different developmental stages in *B. mucronatus*. Tubulin is the basic structural unit of microtubules, which play an important role in maintaining cytoskeletal structure, cell division, cell motility, and contractile processes (Kopczak et al., 1992). Thus, this gene is stably expressed in cells and commonly employed as a reference gene (Campos et al., 2009; Rentoft et al., 2010). *18S rRNA* is commonly used as a reference gene, and has been reported to be the most stable reference genes in different developmental stages of *Lucilia cuprina* (Bagnall and Kotze, 2010), in different tissues of *Rhodnius prolixus* (Majerowicz et al., 2011), in virus-infected *Laodelphax striatella* (Maroniche et al., 2011) and in mammalian cells (Kuchipudi et al., 2012). However, some research has reported that *18S rRNA* may not be appropriate as a reference gene for protein-coding genes, due to its being synthesized by RNA polymerase I, while protein-coding genes are synthesized by RNA polymerase II (Tricarico et al., 2002). Additionally, the expression level of rRNA is much higher than that of protein-coding gene mRNA, while rRNA is far less affected by RNA degradation (Lossos et al., 2003). Indeed, the *C*q value of *18S rRNA* was between 18 and 20 in *B. mucronatus*, while the *C*q value of protein-coding reference genes was more than 20 (**Figure 1**). Our results indicated that several frequently used reference genes such as *ACT* or *PMP* may not be good choices for *B. mucronatus*, especially *ACT*. Under both experimental conditions (different habitat conditions and different developmental stages), the results of the BestKeeper analysis showed that *ACT*, with *SD* > 1, was not suitable as a reference gene (Migocka and Papierniak, 2011). The unstable expression of *ACT* has also been confirmed in other species (Tong et al., 2009; Zhu et al., 2013; Yan et al., 2014). However, a number of previous reports have selected *ACT* as a reference gene in *B. xylophilus* (Qiu et al., 2013; Xu et al., 2015; Deng et al., 2016; Ding et al., 2016), and, to the best of our knowledge, no previous studies have systematically examined its expression stability in *B. xylophilus*. As *B. xylophilus* is a sister species to *B. mucronatus*, and morphological and biological characteristics are very similar in the two species (Mamiya and Enda, 1979), *B. xylophilus* may have arisen from its ancestor *B. mucronatus* in eastern Asia (Kanzaki and Futai, 2002), and further research is needed to elucidate the expression stability of *ACT* in *B. xylophilus*.

The *FAR* protein accelerates the absorption and transportation of fatty acids and retinoids, helping nematodes to infect the host and inhibit the host defenses (Basavaraju et al., 2003). Previous reports demonstrated that *FAR* expression increased when the nematodes infected and colonized host trees (Cheng et al., 2013; Zhou L. et al., 2016). Employing the most suitable gene combination (*TUB*/*18S rRNA*/*UBCE*) to normalize *FAR* expression under the two habitat conditions, we were able to demonstrate that the expression of the gene significantly increased when the nematodes were inoculated into pine trees. However, no significant difference in *FAR* expression was detected when the least stable gene combination (*EF/PMP/ACT*) was used to normalize *FAR* expression in the two habitat conditions. *AK* is a phaosphagen kinase which plays a critical role in energy mobilization in invertebrates (Matthews et al., 2003). The *AK* gene family comprises two members in nematodes (*AK1* and *AK2*), which are differentially expressed at different developmental stages (Kulathunga et al., 2012;Qi et al., 2015). Our results demonstrated the reliability of the *TUB*/*HIS*/*18S rRNA* combination as reference genes to normalize the transcription of *AK1* and *AK2*, as the expression levels of the target genes were dramatically reduced in juveniles (J2 and J3&4), and markedly increased in adults, when compared with eggs. These expression changes were not observed when *ACT/EF/PMP*, the least stable reference gene combination for juveniles, was used for normalization.

## Author Contributions

LZ and FC were responsible for experimental design, data analysis, and manuscript writing. FC and JY were responsible for nematode collections. LZ, JY, and HP were responsible for RNA extraction, PCR, and RT-qPCR.

## Conflict of Interest Statement

The authors declare that the research was conducted in the absence of any commercial or financial relationships that could be construed as a potential conflict of interest.

## Footnotes

1 http://fulxie.fees.us/?type=reference
