# Supplementary material for: Selection of Reliable Reference Genes for RT-qPCR Analysis of Bursaphelenchus mucronatus Gene Expression From Different Habitats and Developmental Stages
Source: Front Genet. 2018 Jul 23;9:269. doi: 10.3389/fgene.2018.00269 (PMC6064934; doi:10.3389/fgene.2018.00269)
Supplement: Supplementary file 1 [file Data_Sheet_1.DOCX]

Supplementary Material

Selection of reliable reference genes for RT-qPCR analysis of *Bursaphelenchus mucronatus* gene expression from different habitats and developmental stages

Lifeng Zhou, Fengmao Chen*, Jianren Ye*, Hongyang Pan

*** Correspondence:** Fengmao Chen: [cfengmao@njfu.edu.cn](mailto:cfengmao@126.com); Jianren Ye: jrye@njfu.edu.cn

# Supplementary Tables

## Supplementary Table1

**Table S1.** Candidate reference gene descriptions.

| **Gene symbol** | **Gene ID** | **Nr-Annotation** | **Gene type** |
| --- | --- | --- | --- |
| *ACT* | CLl56.Contig2 | Actin | Protein coding |
| *EF* | CL1818.Contig2 | Elongation factor 1 alpha | Protein coding |
| *HIS* | CL988.Contig4 | Histone H2A | Protein coding |
| *PMP* | Unigene1780 | Peroxisomal membrane protein | Protein coding |
| *TUB* | CL627.Contig1 | Beta tubulin | Protein coding |
| *UBCE* | Unigene9831 | Ubiquitin conjugating enzyme | Protein coding |
| *UBQ* | CL35.Contig1 | Ubiquitin | Protein coding |
| *18S rRNA* | CL1355.Contig1 | 18S ribosomal RNA | rRNA |

## Supplementary Table2

**Supplementary Table S2.** Intra-group variation of the 8 candidate reference genes estimated by NormFinder in two different habitat conditions.

|  | *ACT* | *EF* | *TUB* | *HIS* | *PMP* | *UBCE* | *UBQ* | *18S rRNA* |
| --- | --- | --- | --- | --- | --- | --- | --- | --- |
| Trees | 0.917 | 0.319 | 0.026 | 0.274 | 1.247 | 0.135 | 0.182 | 0.035 |
| Fungi | 0.440 | 0.0481 | 0.009 | 0.775 | 1.437 | 0.793 | 1.615 | 0.002 |

# Supplementary Figures

## Supplementary Figure 1


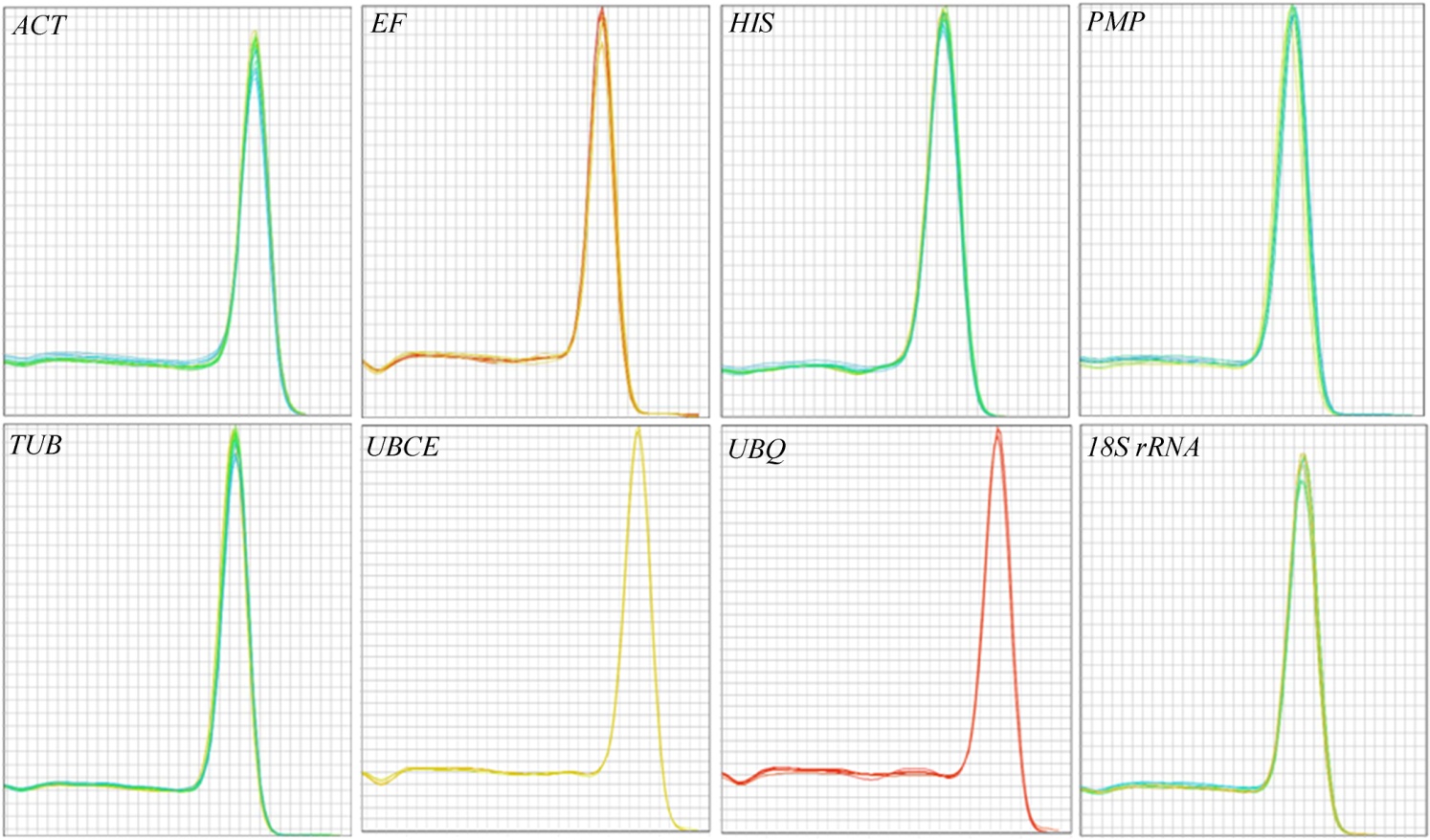


**Supplementary Figure S1.** Melt curves of eight candidate reference genes.

## Supplementary Figure 2


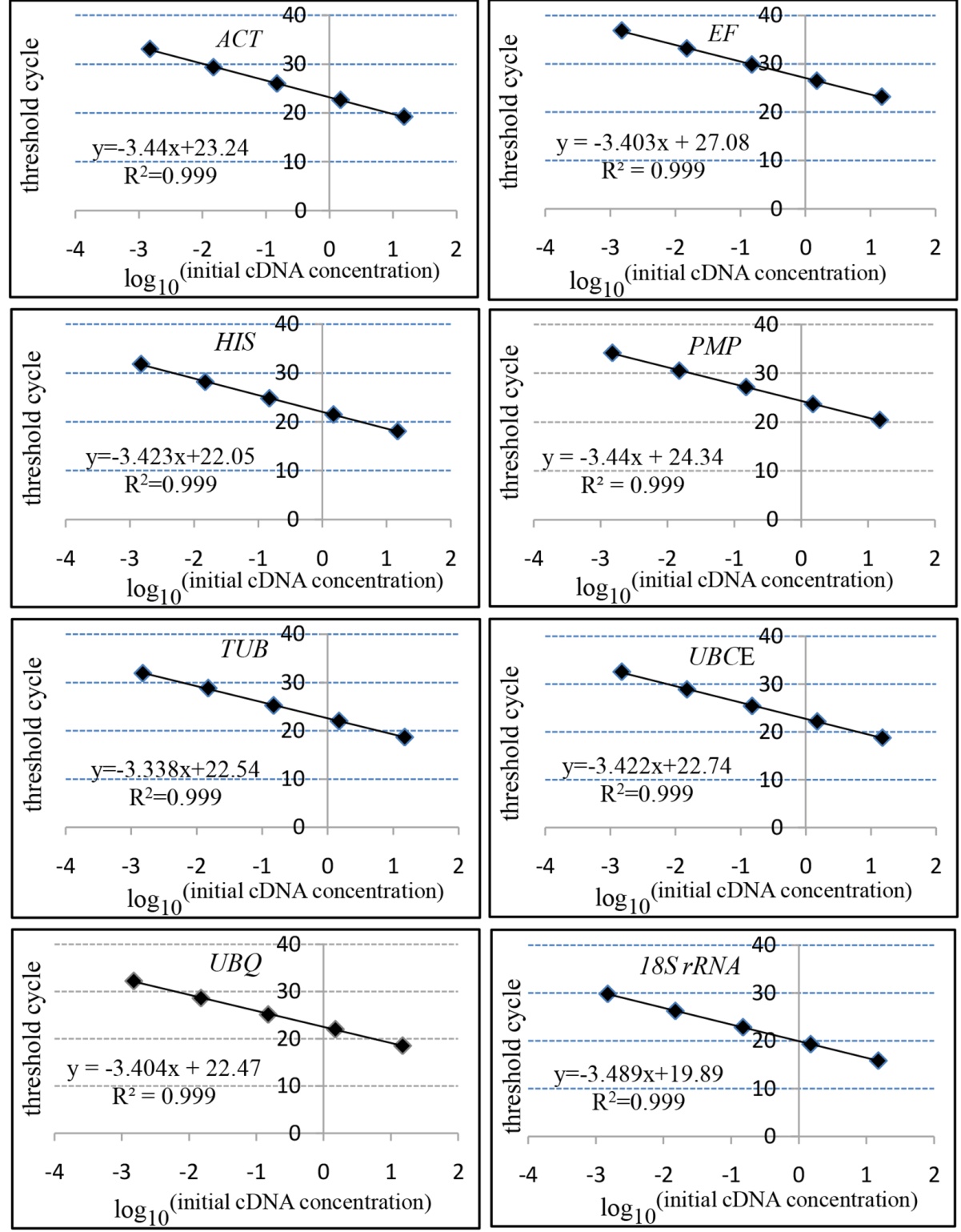


**Supplementary Figure S2.** Standard curves of eight candidate reference genes. Line relations and correlation coefficient (R^2^) between Cq values and Log quantity were calculated by SigmaPlot 12.0 software.
